# Daytime variation in aortic valve surgery: would the chronotype change the end of the story?

**DOI:** 10.5935/1984-0063.20200043

**Published:** 2021

**Authors:** Miguel Meira e Cruz

**Affiliations:** 1 Centro Cardiovascular da Universidade de Lisboa, Lisbon School of Medicine, Sleep Unit - Lisboa - Portugal.; 2 Faculdade São Leopoldo Mandic, Laboratory of Neuroimmune Interface of Pain Research - Campinas - São Paulo - Brazil.

Chronobiology has been recently integrated on several medical fields. Circadian physiology, in particular, was shown to affect several physiological, physiopathological, and pharmacological aspects, which ultimately impact clinical outcomes on different domains. This was brought to attention by Gotte et al.^1^, in their interesting and recently published article on the diurnal oscillation of aortic valve surgery and respective clinical outcomes. Despite the relevance which general features of the human circadian timing system have on guiding biological course of events on either medicine or surgery, it is well known the complex cross-talk between the central master clock, the peripheral oscillators, and the personal time signature that dictates the individual chronotype^2^. Indeed, chronotype, the behavioral manifest of a personalized internal time should be taken into consideration when trying to confirm such challenging hypothesis as that of the influence of external time cues on clinical and surgical outcomes^3^.

In a recent clinical study testing whether time of the day and chronotype could impact the relationship between heart rate variability in sports performance, Vitale et al.^4^ showed that autonomic modulation depends not only on the time of the day but also from different chronotypes (with evening people having greater levels of autonomic disturbance compared with morning type persons). Facer-Childs et al.^5^ also demonstrated diurnal variations in vascular endothelial vasodilation, which were influenced by this individually based temporal domain. Interestingly, some kind of damage on myocardial tissue was suggested to have a circadian fluctuation on its propensity^6^ but also to be function of the chronotype itself^7^. Hence, it seems that not only the biological mechanisms ticked by the circadian clock may interfere and clinically impact the surgical physiology but also the genetically based response through internal time modulated behaviors may probably affect those derived outcomes. So, an important question can be raised: “Could the chronotype assessment, as well as its clinical integration be able to change the end of the story?”
